# Prediction of Postprostatectomy Biochemical Recurrence Using Quantitative Ultrasound Shear Wave Elastography Imaging

**DOI:** 10.3389/fonc.2019.00572

**Published:** 2019-07-09

**Authors:** Cheng Wei, Yilong Zhang, Hamza Malik, Xinyu Zhang, Saeed Alqahtani, Dilip Upreti, Magdalena Szewczyk-Bieda, Stephen Lang, Ghulam Nabi

**Affiliations:** ^1^Division of Imaging Science and Technology, School of Medicine, Ninewells Hospital, University of Dundee, Dundee, United Kingdom; ^2^School of Science and Engineering, University of Dundee, Dundee, United Kingdom; ^3^Division of Population Health and Genomics, School of Medicine, University of Dundee, Dundee, United Kingdom; ^4^Department of Radiological Sciences, College of Applied Medical Science, Najran University, Najran, Saudi Arabia; ^5^Department of Clinical Radiology, Ninewells Hospital, Dundee, United Kingdom; ^6^Department of Pathology, Ninewells Hospital, Dundee, United Kingdom

**Keywords:** prostate cancer, radical prostatectomy, biochemical recurrence, ultrasound shear wave elastography, nomogram

## Abstract

**Objectives:** To determine the prognostic significance of tissue stiffness measurement using transrectal ultrasound shear wave elastography in predicting biochemical recurrence following radical prostatectomy for clinically localized prostate cancer.

**Patients and Methods:** Eligible male patients with clinically localized prostate cancer and extraperitoneal laparoscopic radical prostatectomy between November 2013 and August 2017 were retrospectively selected. Information of potential biochemical recurrence predictors, including imaging (ultrasound shear wave elastography and magnetic resonance imaging), clinicopathological characteristics, and preoperative prostate specific antigen (PSA) levels were obtained. Recurrence-free survival (Kaplan–Meier curve) and a multivariate model were constructed using Cox regression analysis to evaluate the impact of shear wave elastography as a prognostic marker for biochemical recurrence.

**Results:** Patients experienced biochemical recurrence in an average of 26.3 ± 16.3 months during their follow-up. A cutoff of 144.85 kPa for tissue stiffness measurement was estimated for recurrence status at follow-up with a sensitivity of 74.4% and a specificity of 61.7%, respectively (*p* < 0.05). In univariate analysis, shear wave elastography performed well in all preoperative factors compared to biopsy Gleason Score, PSA and magnetic resonance imaging; in multivariate analysis with postoperative pathological factors, shear wave elastography was statistically significant in predicting postoperative biochemical recurrence, which improved the C-index of predictive nomogram significantly (0.74 vs. 0.70, *p* < 0.05).

**Conclusions:** The study revealed that quantitative ultrasound shear wave elastography-measured tissue stiffness was a significant imaging marker that enhanced the predictive ability with other clinical and histopathological factors in prognosticating postoperative biochemical recurrence following radical prostatectomy for clinically localized prostate cancer.

## Introduction

The primary goal of biomarker/predictive test research in the field of prostate cancer (PCa) is to increase the prediction rate of postoperative outcome and/or biochemical recurrence (BCR). Radical prostatectomy (open or minimally invasive) is an established treatment option for clinically localized PCa. Thirty to forty percent of men show postoperative BCR and require further adjuvant or salvage treatments on follow-up (1, 2). Predicting recurrence would be useful in setting up personalized treatment plans for patients, as well as selecting men for adjuvant treatment following prostatectomy. An improved method of predicting recurrence can help in reducing overtreatment of PCa recurrence and thereby reducing side effects and improve quality of life.

There are several known predictors for BCR following surgical therapy for PCa published in the literature, e.g., age, prostate specific antigen (PSA), biopsy Gleason Score (bGS), clinical stage, pathology Gleason Score (pGS), and other postoperative data (3); however, ultrasound imaging, specifically ultrasound shear wave elastography (USWE), as imaging marker has not been reported.

Ultrasound imaging has been widely applied to guide transrectal and transperineal biopsies in the detection of PCa (4–6). USWE estimation of prostate tissue stiffness has been recently reported with promising diagnostic accuracy in both the detection and characterization of PCa (7–9). We have recently shown that the technology can reliably predict the grade of cancer and may provide essential information on the biology and microenvironment of the cancerous lesions. Moreover, USWE showed a very high diagnostic accuracy in predicting clinically significant PCa in men opting for radical prostatectomy (10, 11).

The aim of this study was to assess the predictive usefulness of USWE measured tissue stiffness in postprostatectomy BCR.

## Materials and Methods

### Study Population

**Figure 1:**
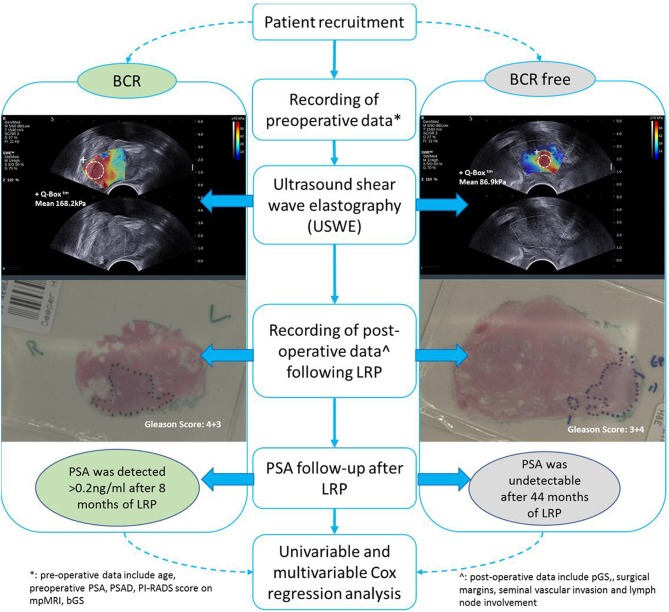
Examples of BCR and BCR-free patients in comparison of SWE and clinicopathology data.

Between November 2013 and August 2017, 212 consecutive men opting for extraperitoneal radical prostatectomy as a treatment option for clinically localized PCa were selected. This was part of a prospective, protocol-driven study with prior ethical and institutional approval [Research Ethical Committee (REC) No. 13/ES/0099, and Research and Development No. 2012ON32] designed to assess the diagnostic accuracy of transrectal SWE ultrasound specifically for PCa. The basic demographic characteristics are presented in Table 1. Men were followed up postoperatively in clinics with PSA measurements every 3 months for the first year, 6-monthly for the second year, and once a year thereafter. A PSA of more than 0.2 ng/ml was considered as a cutoff for consideration of BCR, and this generated a request to discuss the case in multidisciplinary meeting for imaging and adjuvant/salvage treatment. The patient cohort was divided into two groups: BCR and BCR free groups as illustrated in Figure 1. PSA follow-up was recorded until April 2019, and the minimum follow-up for the recruited patients was 21 months.

**Table 1:** Characteristics of recurrence and non-recurrence patients.

	**Total**	**Recurrence**	**Non-recurrence**
No. of patients	209	45	164
Age (years), mean ± SD (range)	66 ± 4.92 (53–76)	66 ± 5.20 (59–76)	66 ± 4.87 (53–76)
Preoperative PSA level (ng/ml), mean ± SD (range)	11.26 ± 7.48 (0.1–47.7)	14.21 ± 9.81 (2–47.7)	10.49 ± 6.56 (0.1–47)
PSA density (ng/ml^2^), mean ± SD (range)	0.19 ± 0.15 (0.001–1.11)	0.26 ± 0.20 (0.05–1.11)	0.17 ± 0.12 (0.001–0.92)
Gleason score at biopsy			
3+3	39 (18.7%)	4 (1.9%)	35 (16.7%)
3+4	76 (36.4%)	8 (3.8%)	68 (32.5%)
4+3	39 (18.7%)	8 (3.8%)	31 (14.8%)
3+5	7 (3.3%)	3 (1.4%)	4 (1.9%)
4+4	27 (12.9%)	9 (4.3%)	18 (8.6%)
4+5	17 (8.1%)	13 (6.2%)	4 (1.9%)
5+3	1 (0.5%)	–	1 (0.5%)
5+4	3 (1.4%)	–	3 (1.4%)
PI-RADS			
Benign	8 (3.8%)	1 (0.5%)	7 (3.3%)
3	17 (8.1%)	1 (0.5%)	16 (7.7%)
4	45 (21.5%)	4 (2%)	41 (19.6%)
5	131 (62.7%)	35 (17%)	96 (45.9%)
N/I^*^	8 (3.8%)	4 (1.9%)	4 (1.9%)
Surgical margin			
Negative	126 (60.3%)	11 (5.3%)	115 (55.0%)
Positive	81 (38.7%)	32 (15.3%)	49 (23.4%)
N/I	2 (1.0%)	2 (1.0%)	–
Gleason Score after RP			
3+3	5 (2.4%)	–	5 (2.4%)
3+4	101 (48.3%)	2 (1.0%)	99 (47.4%)
4+3	34 (16.3%)	7 (3.3%)	27 (12.9%)
3+5	19 (9.1%)	7 (3.3%)	12 (5.7%)
4+4	3 (1.4%)	–	3 (1.4%)
4+5	45 (21.5%)	27 (12.9%)	18 (8.6%)
N/I	2 (1.0%)	2 (1.0%)	–
Vascular invasion			
Negative	185 (88.5%)	31 (14.8%)	154 (73.7%)
Positive	16 (7.7%)	10 (4.8%)	6 (2.9%)
N/I	8 (3.8%)	4 (1.9%)	4 (1.9%)
Lymph node involvement			
Negative	186 (89.0%)	32 (15.3%)	154 (73.7%)
Positive	10 (4.8%)	8 (3.8%)	2 (1.0%)
N/I	13 (6.2%)	5 (2.4%)	8 (3.8%)

* *No information*.

### Clinicopathology Information

All clinical data [age, weight, preoperative PSA and relative density (PSAD), PI-RADS score on multiparametric MRI (mpMRI), bGS, USWE], postoperative data [pGS, surgical margins (SM), seminal vascular invasion (SVI), lymph node involvement (LNI)], and clinical follow-up (months) were recorded. In all eligible patients, PSA fell to an undetectable level (<0.1 ng/ml) postoperatively. BCR was defined as any two consecutive PSA measurements ≥0.2 ng/ml detected during the follow-up (12).

### MR Protocol and PI-RADS Scoring

MRI scan for each patient was carried out 6–8 weeks after the last biopsy with a 3-T scanner (TIM Trio, Siemens, Erlangen, Germany) to eliminate the artifacts due to blood clots caused by biopsy. Then, radical prostatectomy was usually done within 2 months (62 days targets) after MRI in this cohort. MRI protocol was derived from the European Society of Uro-radiology Guidelines (ESUR) 2012 (13) for PCa detection; acquisition parameters are shown and summarized in Table 2 (10, 11). All MR images were analyzed and scored by experienced uro-radiologists (SA, SMB) using PI-RADS v2.0; patients' clinicopathology data were blinded to both radiologists. Only suspicious lesions with PI-RADS score 3 and above were marked. Some of the patients' PI-RADS scores were not available because of inadequate sequences or poor images.

**Table 2:** MRI acquisition parameters.

	**T1WI**	**High-resolution T2WI**			**DWI**		**DCE**
	**Axial**	**Sagittal**	**Axial**	**Coronal**	**DWI**	**DWI high *b* value**	**Dyn Gd-MRI**
Sequence	2DTSE	2DTSE	2DTSE	2DTSE	2DEPI	2DEPI	3D VIBE
TR (ms)	650	6,000	4,000	5,000	3,300	3,300	4.76
TE (ms)	11	102	100	100	95	95	2.45
Flip angle (°)	150	140	150	150	–	–	10
Slice thickness (mm)	3	3	3	3	3	3	3
Slice gap (mm)	0.6	0.6	0.6	0.6	0	0	0.6
Resolution (pixels)	320	320	320	320	192	192	192
FOV (mm)	200	200	200	200	280	280	280
*b* values (s/mm^2^)	–	–	–	–	50, 100, 500, 1,000	2,000	–
Temporal resolution (s)	–	–	–	–	–	–	4

### USWE Imaging Protocol and Acquisition

All USWE images were obtained using a transrectal endocavity transducer (SuperSonic Imagine, Aix en Provence, France) with patients being in either lithotomy or lateral position the day before the scheduled surgery. USWE mode was activated and prostate gland elastograms were obtained from cranial to caudal direction for each lobe of the prostate. All regions were scanned as described in our previous protocol (7). Briefly, each patient's prostate gland was scanned transrectally, and USWE images were acquired in transverse planes from base to apex with a gap of 4 to 6 mm. The most suspicious lesions located in planes were rescanned in gaps as thin as 2–3 mm and reconstructed offline into 3-D images. These suspicious areas were also examined by rotating transducers in different directions to confirm abnormalities and to perform measurements of their sizes. Three stiffness measurements of shear wave speed in m/s or Young's modulus in kPa using pseudo-color map were obtained by three researchers (GN, CW, and DU) independently. The ratio between abnormal and normal areas was also recorded.

### Statistical Analyses

In this paper, BCR as an outcome was a patient-based analysis and hence major predominant and highest histological grade was used. There could be multiple lesions for each patient and lesion-based analysis has been published by our research group previously (10, 11). Baseline characteristics and pathological outcomes were compared using the chi-square test for categorical data and the Student's *t*-test or ANOVA for continuous data. Receiver operator characteristic (ROC) curves were plotted for stiffness values followed by application of maximum Youden index (sensitivity–[1–specificity]), indicating that sensitivity and specificity were equally important to determine optimal cutoff values between BCR and BCR-free patients (14). Univariate and multivariate Cox regression analyses were used to identify factors predictive of BCR. Analyses were performed using SPSS 22 (IBM Corporation, New York, USA). The alpha level was set at 0.05 to determine two-tailed significance. The predicting outcome was evaluated in nomograms and plotted in R 4.4.1. The values of concordance indexes (C-index) were calculated and compared.

## Results

**Figure 2:**
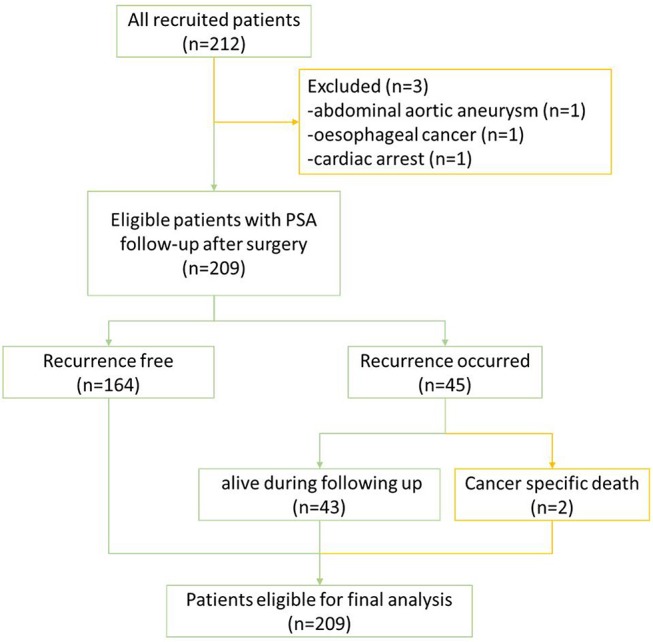
Flowchart of study.

Of all 212 patients, 3 patients were excluded due to death from other diseases during follow-up; the remaining 209 patients eligible for final analysis including 2 patients with PCa-specific deaths (treated as BCR patients) are illustrated in Figure 2. The mean age of the cohort was 66 ± 4.95 years with a mean preoperative PSA of 11.50 ± 7.63 ng/ml (range: 0.1–47.7). Of all the eligible patients, 45 (45/209; 21.5%) experienced BCR in an average of 26.3 ± 16.3 months (range: 3–66) during their follow-up with 25 patients for salvage radiotherapy and 18 patients for hormones therapy. Almost a fifth of participants' biopsy results were GS 6 (39/209, 18.7%), but only 5 (5/209; 2.4%) remained as GS 6 disease on histopathology of radical prostatectomy, suggesting a significant upgrading of disease on surgical resection (Table 1).

**Figure 3:**
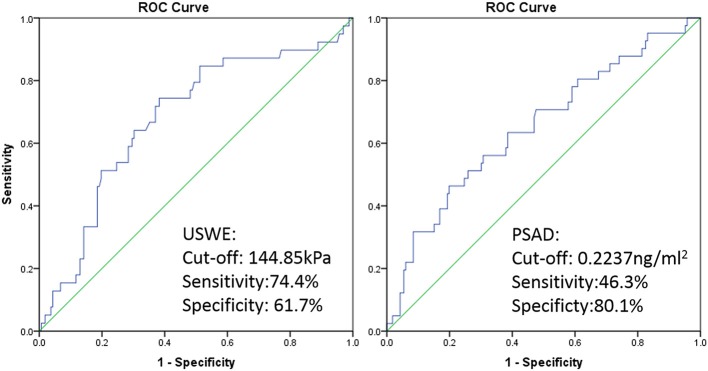
ROC curve of USWE (left) and PSAD (right) for differentiating BCR and BCR free of PCa after radical surgery.

ROC curve was plotted and a cutoff value of 144.85 kPa was calculated with the highest combination of sensitivity and specificity, patients with BCR had 74.4% possibility of cancer stiffness value above 144.85 kPa (sensitivity), and patients with BCR free had a 61.7% possibility of cancer stiffness value below 144.85 kPa (specificity, *p* < 0.05). The cutoff value for preoperative PSAD level differentiating BCR and BCR-free groups was 0.2237 ng/ml^2^ with a sensitivity and specificity of 46.3 and 80.1%, respectively (*p* < 0.05, Figure 3).

On univariate analysis, preoperative PSA level, PSAD, bGS, and USWE were all significantly associated with BCR (*p* < 0.05). Age, weight, and mpMRI using PI-RADS v2 classification without showing any significance (*p* > 0.05) were excluded for further analyses. In the postoperative analysis, pGS was significant in comparison to other parameters in predicting BCR with the highest hazard ratio (HR) of 11.293. Multivariable Cox regression analysis showed the remaining variables excluding LNI (*p* = 0.529) to be statistically significant and independently predictive of BCR (*p* < 0.05, Table 3).

**Table 3:** Cox univariate and multivariate analyses.

		**Univariate**		**Multivariate**	
		**HR (95% CI)**	***P*-value**	**HR (95% CI)**	***P*-value**
Preoperative parameters	Age	1.000 (0.946–1.056)	0.991		
	Weight	0.990 (0.978–1.003)	0.119		
	SWE				
	≤144.85	1 (referent)	–	1 (referent)	–
	>144.85	4.198 (2.036–8.653)	<0.001	3.480 (1.654–7.322)	0.001
	Gleason score at biopsy				
	≤7	1 (referent)	–	1 (referent)	–
	>7	3.919 (2.119–7.248)	<0.001	2.304 (1.207–4.398)	0.011
	PSA level (ng/ml)				
	≤14.45	1 (referent)	–	1 (referent)	–
	>14.45	2.954 (1.576–5.538)	<0.001		
	PSAD (ng/ml^2^)				
	≤0.2237	1 (referent)	–	1 (referent)	–
	>0.2237	2.820 (1.526–5.212)	0.001	2.405 (1.271–4.549)	0.007
	PI-RADS				
	≤3	1 (referent)	–	1 (referent)	–
	>3	2.856 (0.688–11.855)	0.148		
Postoperative parameters	Surgical margin				
	Neg	1 (referent)	–	1 (referent)	–
	Pos	4.930 (2.468–9.848)	<0.001	2.383 (1.136–4.999)	0.022
	Vascular invasion				
	Neg	1 (referent)	–	1 (referent)	–
	Pos	6.020 (2.914–12.434)	<0.001	2.798 (1.236–6.333)	0.014
	Gleason Score at RP				
	≤7	1 (referent)	–	1 (referent)	–
	>7	11.293 (5.199–24.528)	<0.001	6.518 (2.867–14.820)	<0.001
	Lymph node involvement				
	Neg	1 (referent)	–	1 (referent)	–
	Pos	6.044 (2.652–13.775)	<0.001	1.353 (0.528–3.467)	0.529

**Figure 4:**
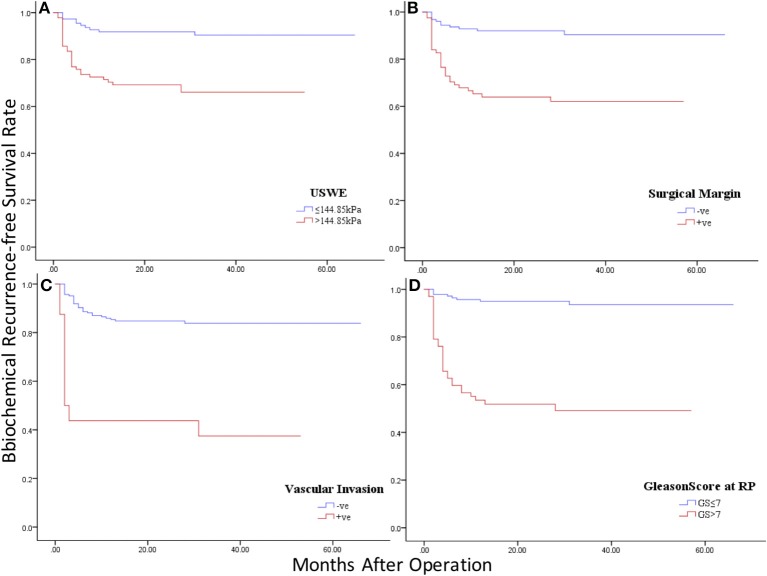
Kaplan–Meier curves of BCR-free survival in four significant predictors: **(A)** ultrasound shear wave elastography (USWE); **(B)** surgical margin (SM); **(C)** seminal vascular invasion (SVI); **(D)** pathology Gleason Score (pGS).

Figure 4 showed Kaplan–Meier survival analysis of BCR-free survival for the four significant predictors including the only preoperative parameter USWE and three postoperative parameters: SM, SVI, and pGS.

**Figure 5:**
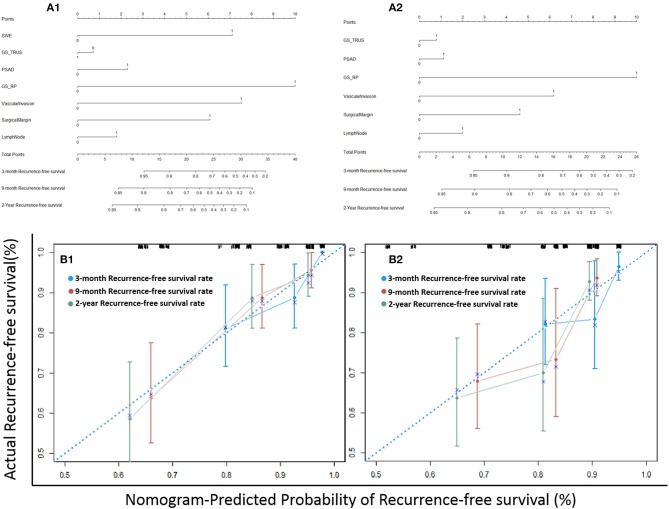
The nomograms of postoperative BCR prediction with **(A1)** and without USWE **(A2)**. Calibration plots of actual and nomogram-predicted probability of BCR with **(B1)** and without USWE **(B2)**.

Figures 5A1,A2 showed the nomograms constructed for BCR with and without USWE data. Longer scales indicated a higher percentage of impact and larger points were followed by shorter BCR survival. The pGS had the greatest impact followed by USWE and SVI. USWE was the only preoperative factor with a significant impact on BCR-free survival. The C-index of the established nomogram that had USWE variate to predict the recurrence-free survival of patients in the cohort was significantly higher than that of the nomogram without USWE {0.747 [95% confidence interval (CI), 0.670–0.824] vs. 0.702 [95% CI, 0.625–0.779], *p* < 0.05}. The nomograms were then internally validated using 100 bootstrap samples; internal calibration curves were shown in Figures 5B1,B2.

## Discussion

To our knowledge, this was the first report to predict BCR with a non-histopathological independent imaging marker—USWE. Clinical and histopathological variables on multivariate analysis have all been previously reported. Introducing USWE into nomograms for PCa BCR with other clinical, pathological, and radiological parameters have increased the accuracy of BCR prediction (0.747 vs. 0.702).

Previous published studies have reported USWE to have a high diagnostic accuracy in clinical staging and localization of tumors within prostate glands of men suspected of PCa (6, 7, 10, 11, 15, 16). Our findings in this study add to the existing knowledge and show that measurement of stiffness in kPa increase BCR prediction ability of clinicopathological parameters.

In this study, the USWE stiffness value was able to distinguish BCR and BCR-free sub-cohort populations as shown in the first ROC curve (Figure 3, left), and then using Youden analysis (14), cancers' stiffness cutoff value 144.85 kPa in Young's modulus was higher in our study in comparison to the number of other reports in diagnosing cancer, but this was defined with the highest combination of sensitivity and specificity, and a larger number of high-grade cancers might have influenced this cutoff value. In this study, we have then added this figure to other clinical and histopathological biomarkers in nomograms for BCR prediction (Figure 5).

Previous studies have analyzed the importance of preoperative and postoperative data in predicting BCR. Kattan et al. (17, 18) and Cooperberg et al. (19, 20) analyzed both data and found that postoperative C-index values were higher than preoperative values (0.89 vs. 0.79, 0.76 vs. 0.66). Grossfeld et al. (21) found PSA levels, bGS, and percentage of biopsy cores involved by cancer tissue to be significant preoperative predictors of BCR. Stephenson et al. (22) validated two groups of patients and had summarized similar postoperative C-index value (0.81 vs. 0.79). Ozden et al. (23) recruited 305 patients and concluded that age was not a significant factor to predict BRC, which was similar to our study. Freedland et al. (24) found that the percentage of biopsy cores involvement by cancer was a better predictor than the number of cores involved by cancer. Similar to these studies, in our observation, PSA and bGS were both significantly important predictors of BCR. The definitions of BCR were varied for different studies as shown in Table 4; this should be considered when comparing results among different studies.

**Table 4:** Literature review and comparison between previous and current studies.

**Author**	**Year**	**Definition of BCR**	**Recruitment**	**Model**	**Predictors**	**C-index**
Kattan et al. (17, 18)	1998	PSA value of 0.4 ng/ml or greater	983	Preoperative	PSA, bGS, clinical stage	0.79
	1999		996	Postoperative	PSA, pGS, SM, SVI, LNI	0.89
Grossfeld et al. (21)	2003	2 consecutive PSA values over 0.2 ng/ml	547	Preoperative	PSA, bGS, ethnicity, percent of positive biopsies	–
Stephenson et al. (22)	2005	PSA value of 0.4 ng/ml or greater	1,782	Postoperative Validation 1	PSA, pGS, ECE, SM, SVI, LNI, treatment years	0.81
			1,357	Postoperative Validation 2		0.79
Cooperberg et al. (19, 20)	2005	2 consecutive PSA values over 0.2 ng/ml	1,439	Preoperative	PSA, bGS, clinical stage, age	0.66
	2011		3,837	Postoperative	PSA, pGS, SM, ECE, SVI, LNI	0.76
This study	2019	2 consecutive PSA values over 0.2 ng/ml	212	Without USWE	PSA, GS, SM, SVI, LNI, USWE	0.702
				With USWE		0.747

MRI characterization of PCa as the most advanced imaging method has been assessed as a potential predictor of BCR as well: Park et al. (25) concluded that PI-RADS might be useful compared to other surgical parameters in 158 patients involved in the study; Tan et al. (26) found that only MRI-detected tumor volume was a significant predictor of BCR. Interestingly, and similar to our findings, PI-RADS v2 was found to be statistically insignificant on univariate analysis and was excluded from further multivariable analysis. Despite its limited contribution as a prognostic factor, mpMRI still remains a key role in PCa detection, staging, and characterization (27, 28).

In the present series, PCa-specific mortality is very low despite a relapse rate of 19% at a short median follow-up of 26.3 months. However, most biochemical relapses following radical prostatectomy will take place within the first 24 months of follow-up, and therefore, studies do give some window of observation. Information from USWE-measured tissue stiffness may potentially inform the decision to enroll patients with a high risk of BCR into clinical trials after considering other factors such as overall health and benefits of the interventions. Using the current data in combination with other clinicopathological factors, it is possible to identify patients at high risk of BCR following prostatectomy, and these men may need an aggressive approach using multimodality approach of combining hormones and chemotherapy particularly when the latter has already shown benefits in metastatic disease (29).

The current study has several limitations: firstly, this is a single institutional study and needs to be externally validated; the application of USWE in clinic still needs additional training and appropriate literature to support it (30). Secondly, there aren't many patients involved with low-risk disease buy may require a long follow-up to know biochemical outcomes. Lastly, as there are a significant number of high-risk patients in this study, a mean of 2 years of follow-up may cover the time period of early biochemical failure in these men, but long-term follow-ups are still needed.

Our findings in this study add to the existing knowledge and show that USWE has the potential ability in a wide clinical application field. This quantitative USWE imaging method may optimize treatment decisions for patients with localized PCa. Further studies are also needed to use this imaging method in accurately selecting cancer patients for adjuvant treatment following radical prostatectomy.

## Data Availability

The datasets generated for this study are available on request to the corresponding author.

## Ethics Statement

This study was carried out in accordance with the recommendations of the East of Scotland Research Ethics Committee 1 (13/ES/0099) and Research and Development (No. 2012ON32) with written informed consent from all subjects. All subjects gave written informed consent in accordance with the Declaration of Helsinki. The protocol was approved by the East of Scotland Research Ethics Committee 1.

## Author Contributions

GN and CW contributed to the study conception and design. GN, CW, DU, MS-B, and SL contributed to the acquisition of data. HM, SL, and SA analyzed the data. CW, YZ, and XZ interpreted the data. CW drafted the manuscript. YZ, HM, XZ, SA, DU, MS-B, and SL contributed to the revision. GN contributed to the critical revision.

### Conflict of Interest Statement

The authors declare that the research was conducted in the absence of any commercial or financial relationships that could be construed as a potential conflict of interest.
